# Impacts of Modified Graphite Oxide on Crystallization, Thermal and Mechanical Properties of Polybutylene Terephthalate

**DOI:** 10.3390/polym13152431

**Published:** 2021-07-23

**Authors:** Hongyan Li, Zhijun Wei

**Affiliations:** 1 Beijing Institute of Technology, Beijing 100081, China; chipsme@126.com; 2 Xi’an Modern Chemistry Research Institute, Xi’an 710065, China

**Keywords:** polybutylene terephthalate, graphene oxide, surface modification, mechanical properties

## Abstract

In this study, the surface modification on graphene oxide (GO) was performed using octadecylamine (ODA). Furthermore, polybutylene terephthalate/GO (PBT/GO) composites were prepared to elucidate the role of GO surface modification on the mechanical performance, thermal stability and crystallization behavior. Results of Fourier transform infrared spectra (FT-IR), Raman spectrum, thermogravimetric analysis (TGA), X-ray photoelectron spectroscopy (XPS) and transmission electron microscope (TEM) revealed that ODA was successfully grafted on GO. Differential scanning calorimetry (DSC), wide-angle X-ray diffraction (WAXD), tensile test, Izod impact strength test and TGA were carried out on the PBT/GO composites. Results indicated that the addition of raw GO can enhance the crystallization temperature and degree of crystallinity and can slightly improve the thermal stability and tensile strength of the composites. However, the impact strength and elongation at break were seriously decreased owing to the poor compatibility between the GO and PBT matrix. Once the modified GO was added, the crystallization temperature and degree of crystallinity were greatly increased. The tensile strength increased greatly while the elongation at break and Izod impact strength were efficiently maintained; these were evidently higher than those of PBT/raw GO. Moreover, thermal stability was greatly enhanced. SEM (scanning electron microscope) observation results on the impact-fractured surface clearly confirmed the improved compatibility between the modified GO and PBT matrix. A related mechanism had been discussed.

## 1. Introduction

Polybutylene terephthalate (PBT) has excellent mechanical, chemical and electrical properties [1]. Due to its excellent comprehensive performance, it is one of the five most commonly used plastics and is widely used in the fields of electronics and electrical appliances, automotive industry and machinery, instruments and household appliances, etc. [2]. To meet the increasingly high demand for different properties for various applications, PBT can be more versatile when it is modified by different additives [3]. For example, the strength and toughness modifications are of great importance, and have received great attention by researchers in recent years [4,5].

Graphene is a two-dimensional carbon nanocrystalline material [6], which is flaky in hexagonal honeycomb lattice and consists of *sp*^2^ hybrid carbon atoms [7,8,9]. The bond connections between carbon atoms are flexible and can bend to adapt to external impact, so its structure is stable. In addition, the tensile modulus of graphene is similar to that of single-walled carbon nanotubes (SWCNTs), with lightweight and large specific surface area and high thermal conductivity [10]. Thus, graphene has been widely used and studied in biochemistry, new materials, electronics, energy and many other fields. Graphene oxide (GO) is oxidized from graphene [11,12,13]. Its surface is rich in different types of oxygen-containing functional groups, thus making it hydrophilic [14]. At the same time, the base surface contains many non-oxidized aromatic regions. Therefore, GO has better amphiphilicity than graphene [15].

However, the special structure of GO brings its excellent properties as well as some difficulties to its application in the field of polymers [16,17]. That is, the large specific surface area and Van der Waals forces between layers of GO lead to its poor dispersion [18,19]. Modification of GO to improve the dispersion of GO in polymer matrix has been a hot choice for many researchers. In recent years, the studies that modify GO by polymer and prepare the GO/polymer composites have gained much attention [6,16,20,21,22]. This is mainly because, compared with other methods, the composites prepared by this method have greater performance improvement. The framework of GO is polycyclic aromatic hydrocarbons, which is relatively stable, so GO is mainly modified by chemical reactions of epoxy, hydroxyl and carboxyl groups on the surface [17]. Carbon skeleton functionalization mainly reacts with C=C bonds of aromatic rings, such as diazo reaction and Diels–Alder reaction [6]. Hydroxyl functionalization mainly uses acyl halide or isocyanate to react with hydroxyl to form esters. The functionalization of epoxy group mainly involves the nucleophilic ring-opening reaction with organic molecules with amino or mercapto groups [23]. The functionalization of carboxyl group is first activated and then dehydrated with amino or hydroxyl groups to form amide or ether bonds [6].

For PBT, previous studies revealed that the addition of graphite or GO could improve the crystallinity and strength of PBT, and the determining role was the compatibility between the filler and the matrix [1,2,3,24,25]. Bian et al. [24] successfully prepared a series of nanocomposites based on PBT and microwave-exfoliated graphite oxide nanosheets (MEGONSs) via melt-compounding technique, and studied the structures, thermal stabilities, mechanical, rheological and electrical properties of the composites. Yang et al. [3] studied prepared high-performance PBT nanocomposites via consideration of phosphorus-containing agents and amino-carbon nanotube (A-CNT). The one-pot functionalization method has been adopted to prepare functionalized CNTs via the reaction between A-CNT and different oxidation state phosphorus-containing agents. Desired compatibility between matrix and the filler was obtained. Shi et al. [25] prepared a series of PBT nanocomposites with polyethylene glycol methacrylate (PEGMA)-functionalized graphene oxide (GO) nanofillers (G-P) as the interfacial modifiers. They reported that PBT/G-P nanocomposites demonstrate good compatibility and interfacial interaction based on the uniform dispersed G-P nanofillers influenced by the grafted PEGMA chains. Their study provided a possible way to prepare high-performance PBT nanocomposites through the enhanced interfacial layer and nucleation behavior of polymer matrix. Wu et al. [2] prepared PBT/graphite (PBT/GP) composites using melt-mixing method. To enhance compatibility between PBT and GP, acrylic acid-grafted PBT (PBT-g-AA) and multi-hydroxyl-functionalized graphene oxide (GO-OH) were used to replace PBT and GP, respectively. They reported that GO-OH could be incorporated into PBT-g-AA copolymer through the formation of strong covalent bonds produced from the reaction between the AA groups of PBT-g-AA and hydroxyl groups of GO-OH.

Octadecylamine (ODA) is commonly used for the surface modification of inorganic fillers, which produces desired effects in many cases [6,26]. However, the impact of ODA surface modification of GO on the mechanical properties, crystallization and thermal stability has not been reported yet. In this paper, the compatibility of GO and PBT is improved by grafting modification of GO with ODA. The chemical structure of the grafted GO has been carefully characterized, and the impact of ODA surface modification of GO on the mechanical properties, crystallization and thermal performance of PBT/GO composites has been comparatively studied, so as to provide a novel method to prepare high-performance PBT composites.

## 2. Experimental Section

### 2.1. Materials

PBT resin with a trade name of L2100G was purchased from SINOPEC Yizheng Chemical Fiber Corp., Ltd. (Yizheng, China). GO was purchased from Guangzhou Angstron Graphene Technology Corp., Ltd. (Guangzhou, China). *N*, *N*-dimethylformamide (DMF) with molecular weight of 73.09, ethanol, dichloromethane, trifluoroacetic acid and octadecylamine (ODA) were purchased from Chengdu Changlian Chemical Reagent Corp., Ltd. (Chengdu, China), and was used as received.

### 2.2. Sample Preparation

#### 2.2.1. Surface Modification of GO

The grafting of ODA onto GO was performed according to the following procedures: GO (0.5 g) and ODA (0.9 g) were stirred and dispersed in 200 mL ethanol for 5 min, then the mixture was stirred (IKA D2025W, IKA Corp., Staufen, Germany) and refluxed at 90 °C for 24 h, and then vacuum filtered to obtain the powder, which was dissolved in 200 mL ethanol again for 5 min, and then vacuum filtered. The washing filtration process was repeated 5 times to eliminate excessive or physically adsorbed ODA. Finally, GO grafted with ODA was dried in a vacuum oven (DHG-9070A, Yetuo Technology Co., Ltd., Shanghai, China) at 60 °C for 24 h.

**Scheme 1:**
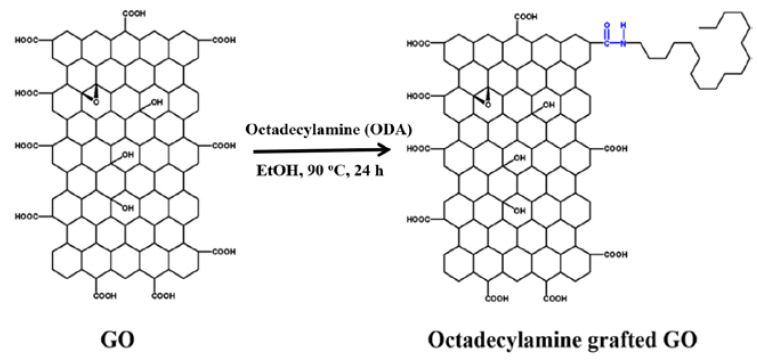
Schematic illustration of grafting of ODA onto GO.

Reaction between ODA and GO are illustrated in Scheme 1. One end of ODA is a long chain of alkanes with 18 carbon atoms, while the other end is a primary amine group. The surface of GO contains a carboxyl-terminated group (-COOH) and an epoxy group (C-O-C). Under certain conditions, affinity addition ring-opening reaction between the primary amine end group (-NH_2_) of ODA and the epoxy group (C-O-C) of GO takes place, and two kinds of groups, C-OH and C-NH-(CH_2_)_17_-CH_3_, can be obtained. Meanwhile, the -NH_2_ group of ODA also reacts with epoxy group carboxyl-terminated group (-COOH) of GO in the manner of amidation reaction [6,27,28].

#### 2.2.2. Preparation of PBT/GO Composites

The preparation of PBT/GO composites was performed according to the manner proposed by a previous study [25]. Raw GO or GO-ODA was first dispersed in a mixed solvent of dichloromethane and trifluoroacetic acid (1:3) via ultrasonication for 3 h to obtain a homogeneous suspension. Then, PBT was slowly dropped into the solution under stirring. Subsequently, PBT/GO or PBT/GO-ODA mixtures were scratched onto a clean glass plate and then were vacuum-dried at 40 °C until constant mass. The mass fraction of GO or GO-ODA in the composites was 1.0 wt.%.

### 2.3. Characterization of Graphene Oxide

#### 2.3.1. Transmission Electron Microscope (TEM)

A small amount of GO or GO-ODA was dispersed in anhydrous ethanol for 1 h under high-frequency ultrasound. A small amount of dispersed droplets was absorbed on the carbon film supported by copper mesh with a straw and observed after the ethanol was dried [29,30]. The above samples were observed by using TECNAI G2 F20 S-TWIN transmission electron microscope (TEM).

#### 2.3.2. Fourier Transform Infrared Spectra (FT-IR)

The FT-IR spectra of ODA, GO and GO-ODA were measured by Nicolet 6700 infrared spectrometer (Thermo Fisher Scientific Corp., Waltham, MA, USA) in the range of 400–4000 cm^−1^. The samples were prepared by KBr compression method [31,32,33].

#### 2.3.3. Raman Spectrum

The Raman spectrum of the samples was measured by LabRAM HR micro laser Raman spectrometer (Horiba Corp., Paris, France) in the range of 500–3500 cm^−1^. The laser wavelength was 633 nm.

#### 2.3.4. X-ray Photoelectron Spectroscopy (XPS)

X-ray photoelectron spectroscopy (XPS) was performed on Kratos AXIS Supra X-ray photoelectron spectroscopy (Shimadzu Corp., Kyoto, Japan.) with Al Kα (1486.6 eV) as the X-ray source set at 150 W and a pass energy of 30 eV for high-resolution scan [34,35,36,37].

#### 2.3.5. Thermogravimetric Analysis (TGA)

TGA measurement was performed by tracing the quality change of 10 mg samples by TG209F1 thermal analyzer (Netzsch Corp., Selb, Germany). The test atmosphere was N_2_, the heating rate was 10 °C/min, and the temperature range was 35–800 °C [38,39,40,41].

### 2.4. Characterization of PBT/GO Composites

#### 2.4.1. Differential Scanning Calorimetry (DSC)

All the calorimetric experiments were performed with a Mettler Toledo DSC1 (Mettler Toledo Corp., Zurich, Switzerland) differential scanning calorimeter (DSC) under a nitrogen atmosphere (50 mL/min) [42,43,44,45,46]. In total, 3−5 mg of samples was used. First, all samples were heated from 30 °C to 260 °C at 10 °C/min and kept for 5 min to erase any previous thermal histories. Then, they were cooled down to 25 °C at a cooling rate of 10 °C/min, respectively. Finally, they were heated to 260 °C at 10 °C/min. The cooling curves and subsequent heating curves of the samples were recorded. The measurement was carried out three to five times and the averaged results were obtained to ensure accuracy.

#### 2.4.2. Wide Angle X-ray Diffraction (WAXD)

WAXD patterns were recorded with DX-1000 diffractometer, Fangyuan Instrument Co., Ltd. (Dandong, China). The wavelength of CuKα was *λ* = 0.154 nm and the spectra were recorded in the 2*θ* range of 5–40°. The scanning rate was 2°/min, and the scanning step was 0.02°. The crystallite size *L* of each plane of samples was determined from WAXD using the Debye–Scherrer equation [47,48,49,50]:

(1) L=0.9λ/βcosθ

where *λ* is the X-ray wavelength of radiation used, *θ* is the Bragg angle and *β* is the full width of the diffraction line at half maximum (FWHM) intensity measured in radians. The measurement was carried out three to five times and the averaged results were obtained to ensure accuracy.

#### 2.4.3. Tensile Performance

In order to test the mechanical properties, the thin sheet with thickness of 1 mm was prepared by casting in the PTFE mold frame by the solution method described above. Then, it was cut into a sample of 80 mm × 10 mm. Then, the specimen was placed at 25 °C for 24 h. After that, an Instron 5944 uniaxial tensile machine with 100 N load cell and a constant 50 mm/min speed was used to perform the tensile tests at 25 °C. For each sample, five to eight specimens were tested and the averaged results with error bar were provided.

#### 2.4.4. TGA Measurement

TGA measurement was used to evaluate the thermal stability of PBT and the composites. TGA measurement was performed by tracing the quality change of 10 mg samples by TG209F1 thermal analyzer (Netzsch Corp., Selb, Germany). The test atmosphere was N_2_, the heating rate was 10 °C/min and the temperature range was 35–800 °C.

#### 2.4.5. Scanning Electron Microscope (SEM) Observation

SEM observation was performed on the fractured surface of the samples [51,52,53,54]. Dried and gold-sprayed samples were observed by FEI Inspect F scanning electron microscope (FEI Corp., New York, NY, USA) under 20 kV accelerating voltage.

## 3. Results and Discussions

### 3.1. Characterization of the Modified Graphene Oxide

#### 3.1.1. XPS Analysis

**Figure 1:**
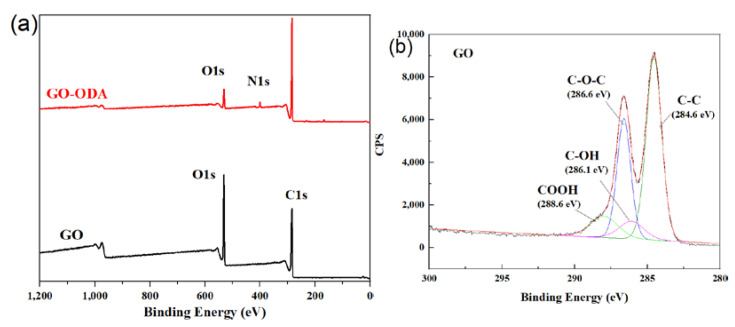

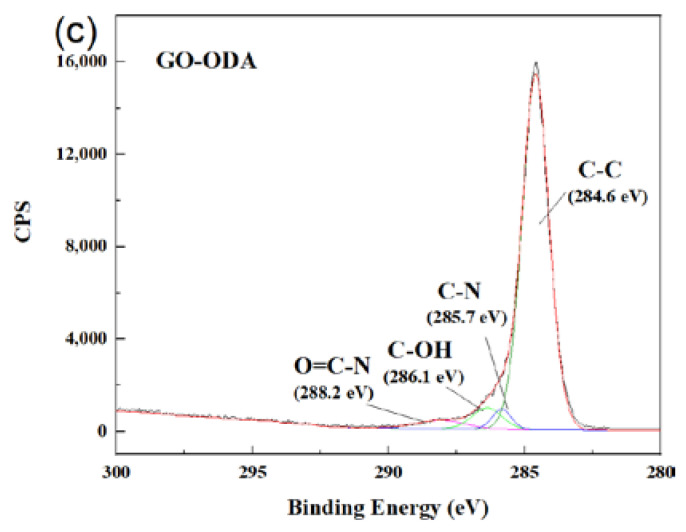
(**a**) XPS profiles of GO and GO-ODA. Peak separation of *C_1s_* of (**b**) GO and (**c**) GO-ODA.

X-ray photoelectron spectroscopy (XPS) can be used to qualitatively analyze the types of chemical bonds on the surface of GO, which is an effective method to characterize the functional groups in GO. Figure 1 shows the XPS spectra and the peak fitting results in C_1s_ of GO and GO-ODA. For GO, the C/O ratio is about 3.1, which indicates that GO is highly oxidized and rich in oxygen-containing functional groups. For GO-ODA, the C/O ratio is about 11.4, the addition of N element is about 3%, and the new N-C=O and C-N bonds are formed by the reaction of ODA with carboxyl and hydroxyl groups on the surface of graphene oxide, which indicates that the alkyl segments are introduced by covalent bonds.

#### 3.1.2. FT-IR, Raman and TGA Analysis

**Figure 2:**
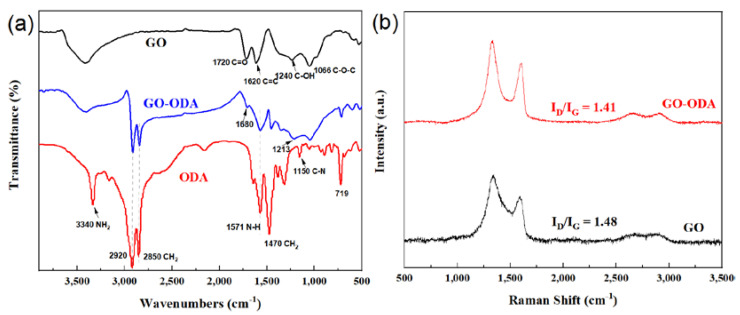
(**a**) FT-IR spectrum of ODA, GO and GO-ODA. (**b**) Raman spectrum of GO and GO-ODA.

This is a very effective method to judge whether the grafting modification of GO is successful or not by FT-IR analysis as shown in Figure 2a. As can be seen from the FT-IR spectrum of GO, ODA and GO-ODA in Figure 2a, for GO, peaks at the wavenumber range of 3000–3700 cm^−1^ represent the stretching vibration of -OH, 1720 cm^−1^ is the stretching vibration of C=O of carboxyl group, 1620 cm^−1^ is the stretching vibration of C=C in benzene structure, 1240 cm^−1^ is the stretching vibration of C-OH and 1066 cm^−1^ is the stretching vibration of C-O-C. The results indicate that GO has at least four functional groups, including -OH, -COOH, -C=O, -CH(O)CH-. On the other hand, ODA has characteristic peaks at 3340 cm^−1^, 1571 cm^−1^ and 1150 cm^−1^, corresponding to N-H stretching vibration, N-H bending vibration and C-N stretching vibration, respectively. The peaks at 2920 cm^−1^ and 2850 cm^−1^ are C-H symmetric stretching vibration and asymmetric stretching vibration, the peaks at 1470 cm^−1^ are C-H deformation vibration in methylene and the peaks at 719 cm^−1^ are characteristic peaks of long chain aliphatic hydrocarbons. For the FT-IR spectra of GO-ODA, the wider peaks at 3000–3700 cm^−1^ can be seen compared with those of GO, which might be attributed to -OH and -NH. There are also symmetric stretching vibration and asymmetric stretching vibration of C-H at 2920 cm^−1^ and 2850 cm^−1^. Moreover, there are two new peaks at 1680 cm^−1^ and 1213 cm^−1^, which correspond to stretching vibration of C=O and stretching vibration of C(O)-N in the -C(O)NHR structure of amide, indicating that ODA is successfully grafted to GO surface by covalent bond instead of physical adsorption.

The Raman spectra of GO and GO-ODA are shown in Figure 2b. Raman spectroscopy is a powerful tool for characterizing carbon materials which is mainly used to distinguish ordered and disordered carbon crystal structures. Different carbon materials have slight differences in structure or size. Raman spectroscopy can detect the unique information of vibration form and electronic properties of carbon nanomaterials. The G peak of carbon nanomaterials in Raman spectrum reflects the E_2g_ vibration model of C=C, which is located at 1500–1605 cm^−1^, indicating the ordered carbon-carbon double bond. The D peak at 1250–1450 cm^−1^ is related to the structural defects of GO. The ratio of D peak to G peak (*I_D_*/*I_G_*) is commonly used to characterize the ratio of *sp*^3^ hybrid to *sp*^2^ hybrid carbon atoms of carbon nanomaterials. The larger the *I_D_/I_G_* value is, the more *sp*^3^ hybrid carbon atoms are in GO. As can be seen from Figure 2b, the *I_D_*/*I_G_* of GO is 1.48, since GO has many defects and a large number of functional groups. For GO-ODA, the strength of D peak and G peak changed, the *I_D_/I_G_* value is 1.41, indicating that the introduction of ODA grafting applied effects on the electronic structure of GO.

#### 3.1.3. TEM Observation

**Figure 3:**
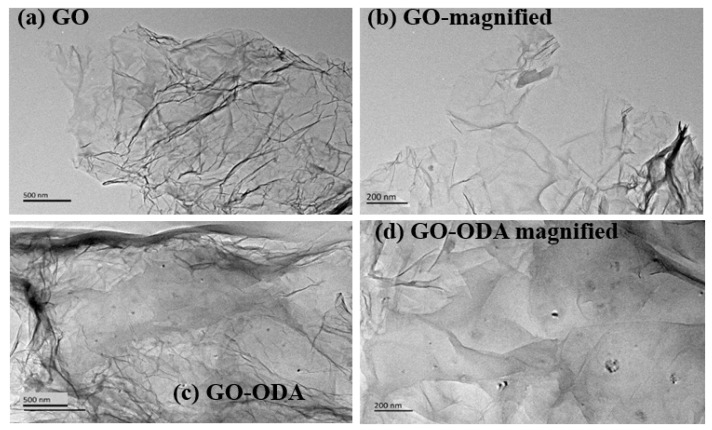
TEM images of (**a**,**b**) GO and (**c**,**d**) GO-ODA at different magnifications.

From the TEM images of GO and GO-ODA shown in Figure 3, it can be seen that flat and smooth GO with a two-dimensional structure is almost transparent. Figure 3b shows the magnified TEM image of raw GO, where one can see thinner and fewer layers on the surface with the unique fold shape and bending phenomenon of GO, indicating that the GO has fewer layers and its performance can be guaranteed. The TEM images of GO-ODA in Figure 3c,d show many dark areas unevenly distributed on the surface of GO, which might be the evidence of ODA grafted on GO surface.

### 3.2. Crystallization Behavior of PBT/GO Composites

#### 3.2.1. DSC Measurement

**Figure 4:**
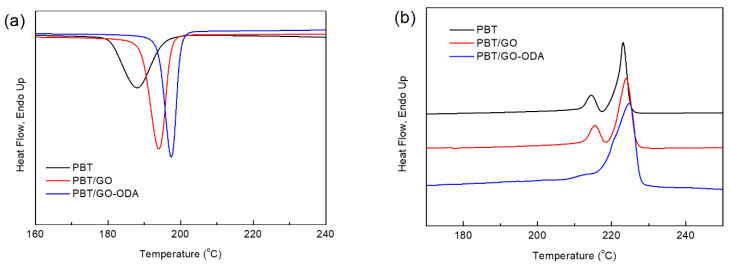
(**a**) Crystallization curves and (**b**) subsequent melting curves of PBT, PBT/GO and PBT/GO-ODA.

The crystallization and melting behaviors of PBT, PBT/GO and PBT/GO-ODA were studied by DSC. The crystallization curves and subsequent melting curves obtained are plotted in Figure 4. The crystallization parameters of the samples were calculated from Figure 4, including the onset crystallization temperature *T_conset_*, crystallization peak temperature *T_cp_*, endset crystallization temperature *T_cendset_* and crystallization peak width *W_cp_*. The melting parameters, including melting points *T_m_*_1_ and *T_m_*_2_ of two melting peaks, were also calculated [55,56]. Furthermore, the relative degree of crystallinity was calculated by the following equation. The crystallization and melting behavior parameters are listed in Table 1 [57,58,59].

(2) $$Xc\;\left(\%,\;Crystallinity\right)=\frac{\Delta H_{m}}{\Delta H_{m}^{{}^{\circ}}\left(1-w_{f}\right)}\times 100\%$$

where Δ*H°_m_* is the melting enthalpy of 100% crystalline PBT (140 J/g), *w_f_* is the mass fraction of filler in composites.

It can be seen from the crystallization curve of the sample (Figure 4a) that the melting peak temperature *T_cp_* of pure PBT is 187.9 °C without the addition of GO, and 193.0 °C after the addition of unmodified go (PBT/GO). When GO-ODA was added, the *T_cp_* of PBT/GO-ODA further increased to 197.5, which was 9.6 °C higher than that of pure PBT, indicating that GO-ODA has better nucleation effect on PBT. At the same time, the onset and endset crystallization temperatures show the same change rule as *T_cp_*. At the same time, the crystallization peak width *W_cp_* of PBT/GO-ODA is 8.8 °C, which is evidently lower than that of PBT/GO (10.6 °C) and pure PBT (16.4 °C). The above results show that GO dispersed in PBT matrix acts as a heterogeneous nucleating agent, promotes the crystallization of PBT, significantly increases crystallization temperature and narrows the crystallization peak width. The addition of GO-ODA can promote the crystallization more obviously. This might be due to the better dispersion of GO modified by ODA in PBT.

On the other hand, it can be seen from the results of melting behavior (Figure 4b) that there are obvious melting double peaks on the melting curves of pure PBT and PBT/GO, which might be caused by the fusion of a certain amount of original crystals, followed by the recrystallization and final melting of more perfect crystals, partly formed during primary crystallization and, through the recrystallization process, occurring during the heating scan. Interestingly, the overlapping of the two peaks of PBT mix forms a new peak with the introduction of GO-ODA, indicating that the presence of GO-ODA improves the crystallization process of PBT and leads to the formation of more perfect and more stable crystals, which melt at higher temperature.

Moreover, the high-temperature peak melting point *T_m_*_1_ of PBT/GO-ODA moves to the high-temperature direction after the addition of GO-ODA, which also shows that the addition of GO-ODA promotes the formation of more perfect and stable crystals. In addition, it can be seen that the relative crystallinity of PBT increases from 36.1% to 38.1% and 40.8%, respectively, after adding unmodified GO and GO-ODA, indicating that the addition of GO can promote more PBT to crystallize, while the effect of GO-ODA is more significant.

#### 3.2.2. WAXD Measurement

**Figure 5:**
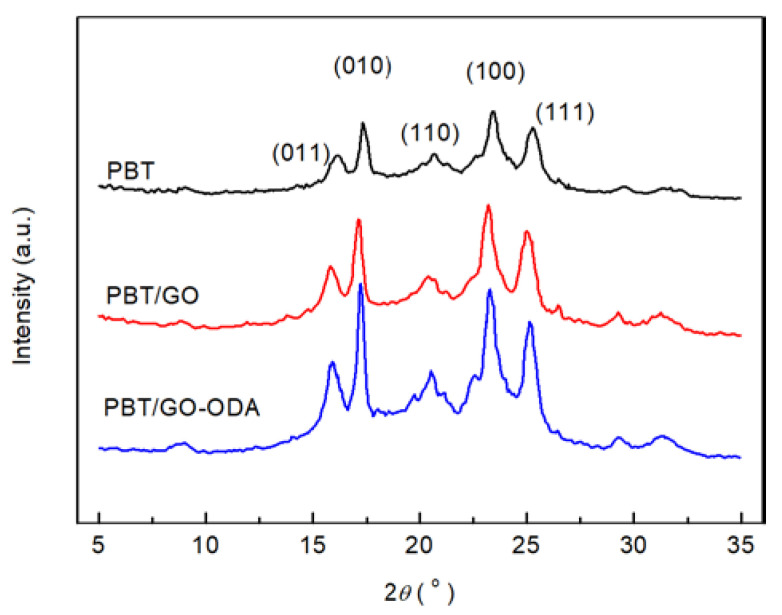
WAXD profiles of PBT, PBT/GO, PBT/GO-ODA and PBT/GO-HBP.

The WAXD patterns of pure PBT and PBT/GO composites are shown in Figure 5. It can be seen from Figure 5 that for all the samples, diffraction peaks are observed at *2θ* Bragg angles of 8.9°, 15.5°, 16.8°, 20.2°, 22.9° and 24.2°, corresponding to diffraction planes (001), (011), (010), (110), (100) and (111), respectively, which are the characteristics of *α*-phase of PBT with triclinic crystal structure. In contrast, PBT/GO shows stronger diffraction peaks, which indicates that the presence of raw GO can promote the formation of α-phase of PBT. For the composites with GO-ODA, the diffraction peak intensity is sharper, indicating that the addition of the filler can promote the crystallization of PBT to a greater extent. In order to obtain quantitative results, the crystallite dimensions *L_hkl_* of each crystal plane are calculated, and the results are listed in Table 2. It can be seen from Table 2 that the crystallite dimensions *L_hkl_* of each crystal plane decrease with the addition of unmodified GO, and the decrease of *L_hkl_* of PBT/GO-ODA is greater compared with that of PBG/GO, indicating that the addition of GO-ODA has a strong nucleation effect on the crystallization of PBT. The increased heterogeneous nucleation sites from GO-ODA affect the growth of PBT crystal, and thus, the adjacent spherulitic morphology is destroyed during the growth process.

### 3.3. Thermal Performance of PBT/GO Composites

**Figure 6:**
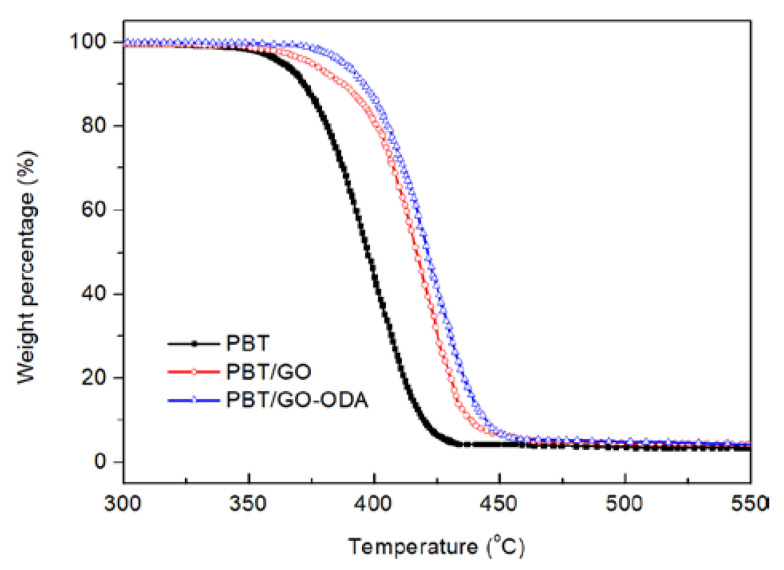
Thermal decomposition curves of PBT, PBT/GO and PBT/GO-ODA.

The thermal decomposition behaviors of neat PBT and its composites under nitrogen condition are shown in Figure 6, and the corresponding data are summarized in Table 3. It is seen that the thermal decomposition behavior of each sample exhibits a one-stage degradation process. According to references, during the thermal decomposition process of PBT, the main volatiles, composed of butadiene, carbon dioxide, tetrahydrofuran, benzoic acid and ester derivatives, are released, leaving behind small solid residues with acidic and anhydride structures [8,9]. For neat PBT, no significant weight loss is observed under 330 °C, and its *T*_1%_ is 342.1 °C. At the temperature above 422.2 °C, it is totally decomposed. When raw GO is added, it can be seen that all the decomposition temperatures in Table 3 increased, indicating that the addition of GO hindered the decomposition and weight loss of the polymer. Interestingly, when GO-ODA was added, the decomposition temperatures of the composites evidently increased. In particular, *T*_1%_ increased 30.8 °C compared with the pure PBT, reflecting that GO-ODA has a higher effect in delaying the thermal decomposition of polymer matrix. This delaying effect of GO and GO-ODA might be attributed to the physical barrier effect. The filler prevents the migration of decomposition products in polymer composites. Similar observations have been made by a previous report [37], where the thermal stability of the polymer/graphene was improved by physical barrier effect and enhanced by ablation recombination of the filler. For GO-ODA, a better dispersion of the filler in the matrix might be the reason for higher enhancement of the thermal stability of the composites.

### 3.4. Mechanical Properties of PBT/GO Composites

**Figure 7:**
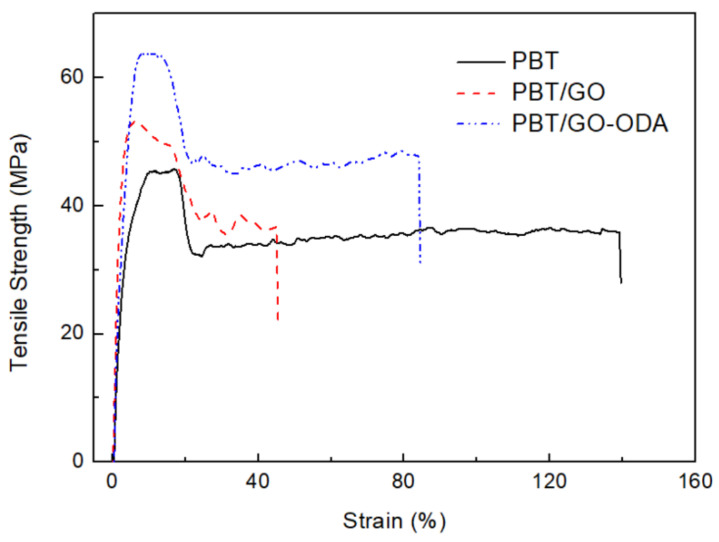
Stress–strain curves of neat PBT and PBT/GO composites.

Tensile test and notched impact test were carried out on PBT and composites. The stress–strain curves are shown in Figure 7, while the obtained parameters, including Izod impact strength, are listed in Table 4. It should be noted that, to ensure the accuracy of the data, each test was repeated five to eight times and then the averaged value with error bar was obtained. Figure 7 and Table 4 show that the tensile strength of PBT/GO composite increases from 45.5 ± 2.4 MPa of pure PBT to 58.6 ± 3.8 MPa; meanwhile, the elongation at break evidently decreases from 136.5 ± 3.5% to 45.1% ± 5.2%, indicating that the dispersion of unmodified GO in PBT might be poor, and the interfacial adhesion between GO and PBT is poor. More evidence can be observed from the stress–strain curves in Figure 7: for PBT/GO, violent fluctuation can be seen from its stress–strain curve, reflecting the weak surface between GO and PBT, which is easy to damage in the tensile test, resulting in the overall failure of the sample, so the elongation at break decreases greatly. Meanwhile, it is observed that GO as inorganic filler can significantly improve the tensile strength of PBT in sacrificing the toughness. For PBT/GO-ODA, the tensile strength and elongation at break are 63.1 ± 2.2 MPa and 81.3 ± 4.4%, respectively, which are significantly higher than those of PBT/GO, indicating that GO surface modification by ODA can significantly improve the compatibility between GO and PBT matrix, leading to higher tensile stress and higher elongation at break.

In terms of impact properties, the impact strength of pure PBT is 7.8 ± 0.8 kJ/m^2^, while the impact strength of PBT/GO evidently decreases to 4.0 ± 1.2 kJ/m^2^, which should also be attributed to poor compatibility between GO and PBT matrix. The impact strength of PBT/GO-ODA is 6.9 ± 0.6 kJ/m^2^, significantly higher than that of PBT/GO, which also reflects that the organic modification on GO surface can significantly improve the compatibility and interfacial adhesion between GO and PBT matrix.

### 3.5. Morphology Investigation (SEM)

**Figure 8:**
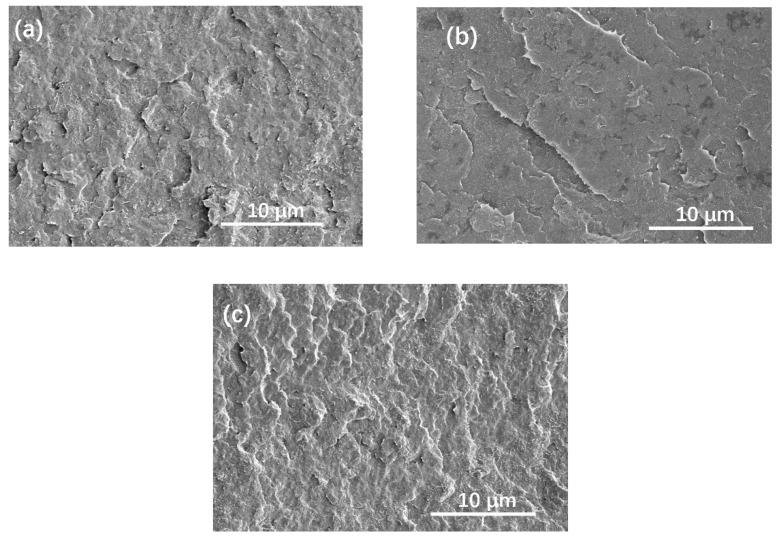
SEM images of the fractured surface of (**a**) PBT, (**b**) PBT/GO and (**c**) PBT/GO-ODA.

To obtain more detailed information, SEM observation was performed on the fractured surfaces of the samples in the impact test. The obtained results are shown in Figure 8.

As can be seen from Figure 8, the morphologies of the fractured surface of the samples are quite different from each other. For pure PBT (Figure 8a), a lot of uneven fracture edges can be seen, which indicates that under the influence of impact stress, owing to the strong bonding force inside the PBT matrix, the impact stress can be well transmitted in a large range of stress damage area as evidenced by the uneven morphology. For PBT/GO, although it is difficult to see the existence of GO from the SEM images, it can be seen that the addition of GO makes the impact fractured morphology change greatly. Generally speaking, the whole impact surface becomes smoother, and the uneven part decreases significantly. This might be due to the fact that the addition of GO leads to the appearance of many weak interfaces between GO and PBT matrix. These weak interfaces tend to deform when subjected to impact stress. Due to the poor interface adhesion between the PBT matrix and GO, it is difficult to transfer the impact stress effectively, resulting in smaller area involved during impact deformation and smoother fracture surface. This is consistent with the fact reported that the impact strength of PBT/GO is significantly lower than that of pure PBT; when GO is modified by ODA, the impact cross-section of the sample turns out to be uneven, and the strain area involved increases significantly under impact stress. This might be attributed to better dispersion of GO in PBT after the ODA surface modification of GO, and the stronger interfacial adhesion between GO and PBT. Therefore, in this case, when the impact stress is applied, the impact stress can be transferred out effectively, resulting in the deformation of a larger area of the sample and the increase of the impact strength.

## 4. Conclusions

In this study, the surface modification on GO surface has been performed using ODA. FT-IR, Raman, TGA, XPS and TEM were used to confirm the success of the grafting. Furthermore, PBT/GO composites were prepared, and the mechanical performances, thermal properties and crystallization behavior of the composites were comparatively studied.

Characterization results on PBT/GO composites revealed that after surface modification of GO using ODA, modified GO can promote the crystallization of PBT to a larger extent, evidently enhancing the tensile strength without sacrificing the elongation at break or impact strength too much, and enhancing the thermal stability of the composites greatly. These enhancements of performances are attributed to improved dispersion and strengthened interfacial interaction between the modified GO and the polymer matrix, revealing that ODA modification of GO can further improve the tensile strength, thermal stability and crystallizability of PBT/GO composites.

## Figures and Tables

**Table 1:** Crystallization and melting parameters of the samples.

Samples	*Crystallization*				*Subsequent Melting*		
	*T_conset_* (°C)	*T_cp_* (°C)	*T_cendset_* (°C)	W_cp_ * (°C)	*T_m1_* (°C)	*T_m2_* (°C)	*X_c_* (%)
PBT	196.7	187.9	180.3	16.4	223.1	214.5	36.1
PBT/GO	198.1	193.0	187.5	10.6	223.8	215.4	38.1
PBT/GO-ODA	201.4	197.5	192.6	8.8	224.8	/	40.8

* *W_cp_* = *T_conset_* − *T_cendset_*.

**Table 2:** Crystalline sizes *L_hkl_* of the samples obtained from WAXD profiles.

Samples	*L_hkl_* (Å)				
	(011)	(010)	(110)	(100)	(111)
PBT	82	228	48	109	107
PBT/GO	74	195	37	99	95
PBT/GO-ODA	66	155	29	91	85

**Table 3:** TGA results of neat PBT and PBT/GO composites.

Samples	*T_1%_* (°C)	*T*_5%_ (°C)	*T_10%_* (°C)	*T_30%_* (°C)	*T_max_*_%_ (°C)
PBT	342.1	363.7	372.3	388.0	422.2
PBT/GO	348.0	375.4	388.1	408.4	436.7
PBT/GO-ODA	372.9	387.7	395.7	411.3	441.8

**Table 4:** Mechanical properties of neat PBT and PBT/GO composites.

Samples	Tensile Strength (MPa)	Elongation at Break (%)	Izod Impact Strength (kJ/m^2^)
PBT	45.5 ± 2.4	136.5 ± 3.5	7.8 ± 0.8
PBT/GO	53.6 ± 4.8	45.1 ± 5.2	4.0 ± 1.2
PBT/GO-ODA	63.1 ± 2.2	81.3 ± 4.4	6.9 ± 0.6

## Data Availability

Not applicable.
